# DNA Data Bank of Japan

**DOI:** 10.1093/nar/gkw1001

**Published:** 2016-10-24

**Authors:** Jun Mashima, Yuichi Kodama, Takatomo Fujisawa, Toshiaki Katayama, Yoshihiro Okuda, Eli Kaminuma, Osamu Ogasawara, Kousaku Okubo, Yasukazu Nakamura, Toshihisa Takagi

**Affiliations:** 1 DDBJ Center, National Institute of Genetics, Shizuoka 411-8540, Japan; 2 Database Center for Life Science, Chiba 277-0871, Japan; 3 National Bioscience Database Center, Japan Science and Technology Agency, Tokyo 102-8666, Japan

## Abstract

The DNA Data Bank of Japan (DDBJ) (http://www.ddbj.nig.ac.jp) has been providing public data services for thirty years (since 1987). We are collecting nucleotide sequence data from researchers as a member of the International Nucleotide Sequence Database Collaboration (INSDC, http://www.insdc.org), in collaboration with the US National Center for Biotechnology Information (NCBI) and European Bioinformatics Institute (EBI). The DDBJ Center also services Japanese Genotype-phenotype Archive (JGA), with the National Bioscience Database Center to collect human-subjected data from Japanese researchers. Here, we report our database activities for INSDC and JGA over the past year, and introduce retrieval and analytical services running on our supercomputer system and their recent modifications. Furthermore, with the Database Center for Life Science, the DDBJ Center improves semantic web technologies to integrate and to share biological data, for providing the RDF version of the sequence data.

## INTRODUCTION

The DNA Data Bank of Japan (DDBJ, http://www.ddbj.nig.ac.jp) (1) is a public database of nucleotide sequences established at the National Institute of Genetics (NIG). Since 1987, the DDBJ has been collecting annotated nucleotide sequences as its traditional database service. This endeavor has been conducted in collaboration with GenBank (2) at the National Center for Biotechnology Information (NCBI) and with European Nucleotide Archive (ENA) (3) at the European Bioinformatics Institute (EBI). The collaborative framework is called the International Nucleotide Sequence Database Collaboration (INSDC, http://www.insdc.org/) (4) and the product database from this framework is called the International Nucleotide Sequence Database (INSD).

Within the INSDC framework, the DDBJ Center also services the DDBJ Sequence Read Archive (DRA), BioProject for sequencing project metadata and BioSample for sample information to facilitate the acceptance of large-scale data generated from next-generation sequencing platforms (5–7). The comprehensive resource of nucleotide sequences and associated information complies with the INSDC policy that guarantees free and unrestricted access to data archives (8). In 2016, the advisors of INSDC published an open letter to remind scientists to submit their sequence data to the INSDC (9,10).

In addition, the DDBJ Center services the Japanese Genotype-phenotype Archive (JGA, http://trace.ddbj.nig.ac.jp/jga) in collaboration with the National Bioscience Database Center (NBDC, http://biosciencedbc.jp/en/) of the Japan Science and Technology Agency (5,11). This database stores personal genotype and phenotype data from individuals who have signed consent agreements authorizing data release only for specific research use. The data access is strictly controlled, similar to the data access policy of the database of Genotypes and Phenotypes at the NCBI (12) and the European Genome-phenome Archive at the EBI (13). NBDC provides the guideline and policies for sharing human-derived data (http://humandbs.biosciencedbc.jp/en/guidelines) and also reviews data submission and usage requests.

The DDBJ Center, a part of NIG, is funded as a supercomputing center. Our web services, including submission systems, data retrieval systems, Web API, DDBJ Read Annotation Pipeline, and backend databases are performed on the NIG supercomputer system. The current commodity-based cluster was implemented in 2012 (14).

The present article reports the update of the above services at the DDBJ Center. A highlight is the semantic web services developed in collaboration with the Database Center for Life Science (DBCLS, http://dbcls.rois.ac.jp/en) and the virtualization of annotation pipeline. All resources described here are available from http://www.ddbj.nig.ac.jp and most of the archival data can be downloaded at ftp://ftp.ddbj.nig.ac.jp.

## THE DDBJ ARCHIVAL DATABASES

### Data contents: traditional DDBJ and the DRA

In 2015, most of nucleotide data directly submitted to the DDBJ (3826 times; 75.3%) were made by Japanese research groups, with the remainder originating from Iran (238 times; 4.7%), India (188 times; 3.7%), Thailand (154 times; 3.0%), China (111 times; 2.2%), and other countries and regions (563 times; 11.1%).

Between June 2015 and May 2016, the DDBJ periodical release increased by 10 317 427 entries and 20 978 161 726 base pairs. The periodical release does not include whole-genome shotgun (WGS), large parts of transcriptome shotgun assembly (TSA) or third party data (TPA) files (15). The DDBJ has continuously distributed sequence data in published patent applications from the Japan Patent Office (JPO, http://www.jpo.go.jp) and the Korean Intellectual Property Office (KIPO, http://www.kipo.go.kr/en). The JPO directly transferred its data to the DDBJ, whereas the KIPO transferred its data via an arrangement with the Korean Bioinformation Center. The DDBJ contributed 19.20% of the entries and 12.84% of the total base pairs added to the core nucleotide data of the INSD. A detailed statistical breakdown of the number of records is shown on the DDBJ homepage (http://www.ddbj.nig.ac.jp/breakdown_stats/prop_ent-e.html). In addition to the above data, the DDBJ has released a total of 11 909 516 WGS entries (1694 genomes), 1 505 087 contig/constructed (CON) entries, 1 313 171 TSA entries (18 projects), 786 TPA entries, 6374 TPA-WGS entries (one genome) and 1272 TPA-CON entries as of 27 May 2016.

In the period between June 2015 and May 2016, next-generation sequencing data of 23,974 runs have been registered to the DRA.

Notable datasets released from the DDBJ sequence databases are listed in Table 1. In particular, we accepted and released the latest sequence data of the reference genome of rice (16), with the annotation performed by the Rice Annotation Project (17) that has been anticipated by many researchers.

**Table 1.** List of notable data sets released from the DNA Data Bank of Japan (DDBJ) sequence databases from June 2015 to May 2016

Data type	Organism	Accession numbers for annotated sequences (number of entries)	Accession numbers for raw reads
Genome	Radish (*Raphanus sativus* cv. Aokubi S-h)	WGS: BAOO01000001-BAOO01072909 (72 909 entries) scaffold CON: DF196826-DF236948 (40,123 entries)	DRR012610-DRR012624
	Soybean (*Glycine max* cv. Enrei)	BBNX02000001-BBNX02108601 (108 601 entries)	DRR021740-DRR021744
	Common marmoset (*Callithrix jacchus*)	WGS: BBXK01000001-BBXK01109198 (109 198 entries) scaffold CON: DG000097-DG000120 (24 entries) GSS: LB274659-LB427105 (152 447 entries)	DRR036754-DRR036764
	Rice (*Oryza sativa* Japonica Group cv. Nipponbare)	chromosome: AP014957-AP014968 (12 entries) unanchored: AP014969-AP015011 (43 entries)	n/a
	Hawaiian acornworm (*Ptychodera flava*)	WGS: BCFJ01000001-BCFJ01317432 (317 432 entries) scaffold CON: LD342582-LD560836 (218 255 entries)	DRR027930-DRR027956
	Azuki bean (*Vigna angularis* var. *angularis*)	chromosome: AP015034-AP015044 (11 entries) scaffold: AP015045-AP017294 (2,250 entries)	DRR031705 DRR031878-DRR031883 DRR032984-DRR033067
	Taiwan habu (*Protobothrops mucrosquamatu*)	WGS: BCNE010000001-BCNE011421934 (1 421 934 entries)	DRR049668, DRR049669
		WGS: BCNE02000001-BCNE02167851 (167 851 entries) scaffold CON: LD636650-LD688929 (52 280 entries)	DRR049668, DRR049669
	*Acropora digitifera*	WGS: BACK02000001-BACK02054400 (54 400 entries) scaffold CON: DF970692-DF973111 (2420 entries)	DRR001380-DRR001433
	*Zoysia japonica* cv. Nagirizaki	WGS: BCLF01000001-BCLF01011786 (11 786 entries)	DRR047281-DRR047283, DRR047291
	*Zoysia matrella* cv. Wakaba	WGS: BCLG01000001-BCLG01013609 (13 609 entries)	DRR047287, DRR047289
	*Zoysia pacifica* cv. Zanpa	WGS: BCLH01000001-BCLH01011428 (11 428 entries)	DRR047288, DRR047290
	A bacterium that degrades and assimilates PET, *Ideonella sakaiensis*	WGS: BBYR01000001-BBYR01000227 (227 entries)	n/a
	Luminous mushroom (*Mycena chlorophos*)	WGS: BAYG01000001-BAYG01025660 (25 660 entries) scaffold CON: DF837679-DF850034 (12 356 entries)	DRR018497-DRR018504
	Ohi'a lehua (*Metrosideros polymorpha* var. *glaberrima*)	WGS: BCNH01000001-BCNH01036376 (36 376 entries)	n/a
	Matsutake (*Tricholoma matsutake*)	WGS: BDDP01000001-BDDP01088884 (88 884 entries)	n/a
	Common buckwheat (*Fagopyrum esculentum*)	WGS: BCYN01000001-BCYN01387594 (387 594 entries)	DRR046985-DRR046993
	Mushroom (*Hypsizygus marmoreus*)	WGS: BDDV01000001-BDDV01010694 (10 694 entries)	n/a
Transcriptome	Radish (*Raphanus sativus* cv. Aokubi S-h)	n/a	DRR010353-DRR010355 DRR014743-DRR014781
	Soybean (*Glycine max* cv. Enrei)	n/a	DRR031435
	Common house spider (*Parasteatoda tepidariorum*)	IAAA01000001-IAAA01132843 (132 843 entries)	DRR047015-DRR047017
	Ayu (*Plecoglossus altivelis altivelis*)	thrombocyte LA715952-LA738445 (22 494 entries) neutrophil LA738446-LA761178 (22 733 entries) B lymphocyte LA761179-LA777683 (16 505 entries)	DRR024801 DRR025094 DRR024802
	Taiwan habu (*Protobothrops mucrosquamatu*)	IAAC01000001-IAAC01112307 (112 307 entries)	DRR049635-DRR049665
	California harvester ant (*Pogonomyrmex californicus*)	IAAD01000001-IAAD01311730 (311 730 entries)	DRR048539-DRR048582
	Ant (*Formica aquilonia*)	LH381539-LH513652 (132 114 entries)	DRR042077-DRR042082 (DRA003820)
	Ant (*Formica cinerea*)	LH513653-LH652103 (138 451 entries)	DRR042083-DRR042088 (DRA003820)
	Ant (*Formica exsecta*)	LH652104-LH973351 (321 248 entries)	DRR042089-DRR042092 (DRA003820)
	Ant (*Formica fusca*)	LI000001-LI121692 (121 692 entries)	DRR042093-DRR042098 (DRA003820)
	Ant (*Formica pratensis*)	LI121693-LI219804 (98 112 entries)	DRR042099-DRR042104 (DRA003820)
	Ant (*Formica pressilabris*)	LI219805-LI349988 (130 184 entries)	DRR042105-DRR042110 (DRA003820)
	Ant (*Formica truncorum*)	LI349989-LI476587 (126 599 entries)	DRR042111-DRR042116 (DRA003820)
	Ant (*Lasius neglectus*)	LI476588-LI563515 (86 928 entries)	DRR042123-DRR042128 (DRA003820)
	Ant (*Lasius turcicus*)	LI563516-LI670604 (107 089 entries)	DRR042129-DRR042134 (DRA003820)
	Ant (*Linepithema humile*)	LI670605-LI795928 (125 324 entries)	DRR042117-DRR042122 (DRA003820)
	Ant (*Monomorium chinense*)	LI795929-LI926639 (130 711 entries)	DRR042135-DRR042140 (DRA003820)
	Ant (*Monomorium pharaonis*)	LJ000001-LJ120855 (120 855 entries)	DRR042141-DRR042146 (DRA003820)
	Ant (*Myrmica rubra*)	LJ120856-LJ206166 (85 311 entries)	DRR042147-DRR042152 (DRA003820)
	Ant (*Myrmica ruginodis*)	LJ206167-LJ284088 (77 922 entries)	DRR042153-DRR042158 (DRA003820)
	Ant (*Myrmica sulcinodis*)	LJ284089-LJ356044 (71 956 entries)	DRR042159-DRR042164 (DRA003820)
	Red fire ant (*Solenopsis invicta*) monogynous	LJ356045-LJ530869 (174 825 entries)	DRR042165-DRR042170 (DRA003820)
	Red fire ant (*Solenopsis invicta*) polygynous	LJ530870-LJ707314 (176 445 entries)	DRR042171-DRR042176 (DRA003820)
	Tausch's goatgrass (*Aegilops tauschii*)	Strain AT76: IAAN01000001-IAAN01045723 (45 723 entries)	DRR058959
		Strain KU-2003: IAAO01000001-IAAO01055813 (55 813 entries)	DRR058960
		Strain KU-2025: IAAP01000001-IAAP01033680 (33 680 entries)	DRR058961
		Strain KU-2075: IAAQ01000001-IAAQ01065447 (65 447 entries)	DRR058962
		Strain KU-2078: IAAR01000001-IAAR01060884 (60 884 entries)	DRR058963
		Strain KU-2087: IAAS01000001-IAAS01065827 (65 827 entries)	DRR058964
		Strain KU-2093: IAAT01000001-IAAT01053474 (53 474 entries)	DRR058965
		Strain KU-2124: IAAU01000001-IAAU01060479 (60 479 entries)	DRR058966
		Strain KU-2627: IAAV01000001-IAAV01060547 (60 547 entries)	DRR058967
		Strain PI499262: IAAW01000001-IAAW01055848 (55 848 entries)	DRR058968

### Japanese genotype-phenotype archive

The JGA is a permanent archiving service for genotype and phenotype data of human individuals (11). The JGA accepts data that are de-identified by submitters. Upon submission, the JGA team will archive the original data files in encrypted form in the secure database. As of 1 September 2016, the JGA has archived 57 studies (23.5 TB) of individual-level human datasets submitted by Japanese researchers. Archived studies include ‘development of molecular targeted therapy for small cell lung cancer by comprehensive genome analysis’ (18), ‘transcriptome analysis of adolescents and young adults with Acute Lymphoblastic Leukemia’ (19) and ‘Japanese Alzheimer's disease neuroimaging initiative’ (20). Submission of these studies has been reviewed and approved by the Data Access Committee at the NBDC. The summaries of 37 studies are available to the public both on the JGA (https://ddbj.nig.ac.jp/jga/viewer/view/studies) and NBDC (http://humandbs.biosciencedbc.jp/en/data-use/all-researches) websites. To access individual-level data of these public studies, users are required to apply data access requests to the NBDC (http://humandbs.biosciencedbc.jp/en/data-use). The DAC ensures that the stated research purposes are compatible with participant consent and that the principal investigator and institution will abide by the NBDC guideline and the specific terms and conditions imposed to a given dataset. Once access has been granted by DAC, datasets with access permission can be downloaded with a secure software tool. It is required for users to establish a secure computing facility for local use of the downloaded data according to the NBDC security guideline.

## DDBJ SYSTEM UPDATE

### Update registration systems for the DDBJ traditional assembled sequence archives

We provide two systems for data submission to the traditional DDBJ database: (i) the Nucleotide Sequence Submission System (NSSS; 5) and (ii) the Mass Submission System (MSS; 21). The NSSS is an interactive application facilitating the entry of all items via a web-based form, http://www.ddbj.nig.ac.jp/sub/websub-e.html. The MSS is a procedure to directly send large data files, http://www.ddbj.nig.ac.jp/sub/mss_flow-e.html. Both systems were enhanced to apply the new rules of feature and qualifier usages (see http://www.ddbj.nig.ac.jp/insdc/icm2015-e.html#ft). As mentioned above, the data volume of TSA submissions to the DDBJ was dramatically increased, with individual submissions of 100 000 sequences. Therefore, we decided to improve the DDBJ accession number assignment system to accept such bulk TSA submissions. Since October 2015, the DDBJ has assigned accession numbers with four letter prefixes for TSA data submitted to the DDBJ, similar to the WGS data. During November 2015, the DDBJ released TSA data with a four letter prefix IAAA (IAAA01000001–IAAA01132843) for the first time (Table 1). See also the anonymous FTP site of TSA data, ftp://ftp.ddbj.nig.ac.jp/ddbj_database/tsa/.

### Sequence analytical services

#### The NIG supercomputer as a sequence analytical platform

The DDBJ Center operates the NIG supercomputer which specializes in analysis of large-scale sequence data. The NIG supercomputer offers computational infrastructure for the construction of DDBJ databases and analysis services, and provides researchers with a large-scale data analysis and supercomputing environment. The NIG supercomputer is currently composed of two computer systems: (i) the Phase 1 system which was introduced in 2012 and (ii) the Phase 2 system which went into production in 2014. The Phase 1 system consists of calculation nodes for general-purpose (352 thin-nodes, each with 64 GB memory; Intel Xeon E5-2670 5632 cores, 117.14 Tflops peak performance of CPUs in total) and memory-intensive tasks, including *de novo* assembly of sequencing reads: two medium nodes, each with 2 TB of memory (HP DL980G7: Intel Xeon E7-4870 160 cores 1.22 Tflops in total), and one fat node with 10 TB of memory (SGI UV1000: Intel Xeon E7-8837 762 cores, 8.17 Tflops). In the general-purpose thin calculation nodes, 64 thin nodes contain NVIDIA Tesla M2090 GPGPU. The Phase 2 incorporates 202 thin nodes, each with 64 GB of memory (Intel Xeon E5-2680v2 4040 cores, 90 Tflops in total) and eight medium nodes (identical to Phase 1).

The calculation nodes in each system are interconnected with InfiniBand (QDR in Phase 1 and FDR in Phase 2) by a complete bisection fat-tree topology. To support massive I/O in the big-data analysis, the NIG supercomputer is equipped with 7 PB of the Lustre parallel distributed file system (http://www.lustre.org). The 5.5 PB MAID system is used for archiving of the Sequence Read Archive data, including the DRA and JGA (11). The number of NIG Supercomputer users increased from 2016 in 1 June 2015 to 2532 by 31 May 2016. The criteria for issuing a user login account are shown on the web page (https://sc.ddbj.nig.ac.jp/index.php/en/criteria-for-issuing-user-login-accounts).

#### Supported analytical tools and public datasets in the NIG Supercomputer

Many popular bioinformatics tools and libraries were installed in the system for the convenience of the login users of the NIG supercomputer, as listed on the NIG supercomputer home page (http://sc.ddbj.nig.ac.jp/index.php/ja-avail-oss). To help reproduce previously executed analysis flow, different versions of the analytical tools are installed in different directory paths. Pre-installed datasets in the NIG supercomputer for those analytical tools are listed on the web page (http://sc.ddbj.nig.ac.jp/index.php/ja-availavle-dbs).

#### Web BLAST, ClustalW, VecScreen, ARSA and Web API for Bioinformatics (WABI)

The DDBJ Center has provided the Web BLAST (22), ClustalW (23,24) and VecScreen (http://www.ncbi.nlm.nih.gov/tools/vecscreen/univec) services, which receive requests from web interfaces. The DDBJ Center also provides the Web API for Bioinformatics (WABI) (25–27) for large scale data analysis and the RESTful Web API service that can process requests from computer programs. The WABI service includes BLAST, VecScreen, ClustalW, MAFFT (28,29), getentry data retrieval system via accession numbers and the ARSA keyword search system for the DDBJ flat files (14). The WABI service recently incorporated a new feature of MAFFT version 7 (–add, –addfragments, –addprofile, and –addfull options), which allow the addition of unaligned sequences into an existing alignment (29).

#### TXSearch to retrieve NCBI taxonomy index

**Figure 1.**
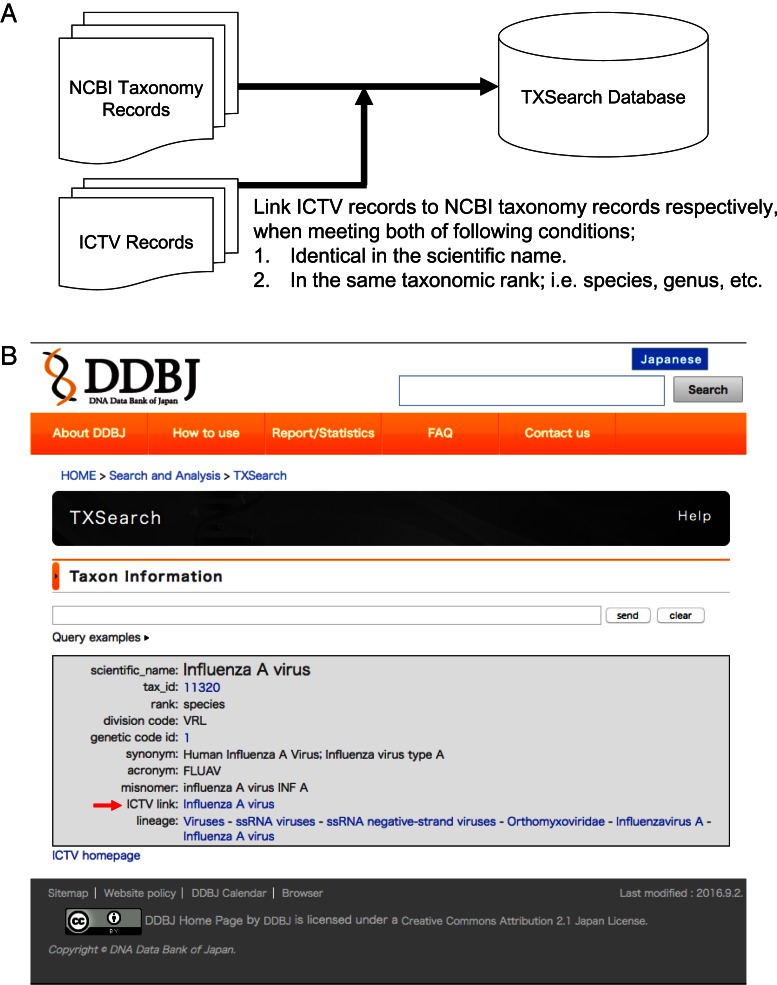
Improvement to link ICTV records on the viral taxonomic records of TXSearch tool. (**A**) Schematic diagram of data flow to insert links to ICTV records into NCBI Taxonomy records. (**B**) Screenshot of a viral record on TXSearch tool. The red arrow shows a link to the ICTV record.

TXSearch (http://ddbj.nig.ac.jp/tx_search/) is an NCBI Taxonomy browsing system in the DDBJ. This browsing system allows data submitters to find authentic scientific names used in the INSDC for the purpose of vocabulary control. Due to the replacement of the NIG supercomputer in 2012, we re-implemented most of our services on open source middleware for accommodation on the new system. The TXSearch system was built on the Apache Solr full text search system and MySQL. The RESTful Web API service is also provided. The data in the TXSearch are updated on a daily basis by downloading the NCBI Taxonomy database (30) from the NCBI FTP site (ftp://ftp.ncbi.nih.gov/pub/taxonomy). Currently, viral records of TXSearch contain links to records of Virus Taxonomy: 2015 Release (31), released from the International Committee on the Taxonomy of Viruses (ICTV http://www.ictvonline.org/) as shown in Figure 1.

#### A virtual machine image for the DDBJ Pipeline

The DDBJ Read Annotation Pipeline (DDBJ Pipeline, http://p.ddbj.nig.ac.jp) is a high-throughput web annotation system of next-generation sequencing reads running on the NIG supercomputer (32). The pipeline's basic component facilitates reference genome mapping and *de novo* assembly, and subsequent components such as structural and functional annotation analyses with a Galaxy interface (33). During 2016, the subsequent component of DDBJ Pipeline was moved from a web service on the NIG supercomputer to a software distribution service for both the local Oracle VirtualBox and Pitagora-Galaxy community web server (http://www.pitagora-galaxy.org/) organized by Dr Ryota Yamanaka. Users are required to operate the virtual machine on their own local environment or flexible cloud computing environment. Thus, computational resources in the NIG supercomputer for the DDBJ Pipeline service was concentrated from both basic and succeeding components into only the basic component, which often requires heavy memory usages and comprises time intensive tasks.

#### Semantic Representation of DDBJ Annotated Sequence Records

To improve reusability of the sequence annotation data, we have developed a system to make the DDBJ records into the Resource Description Framework (RDF) version in collaboration with DBCLS (11,34,35). To semantically represent the DDBJ nucleotide sequence annotation, we have developed a DDBJ taxonomy ontology for describing taxonomic information of the source organism and a DDBJ annotated nucleotide sequence ontology for describing metadata such as submitters and references, and biological feature annotations (http://ddbj.nig.ac.jp/ontologies/). Besides semantic information based on those ontologies, the RDF dataset contains the semantic relations expressed using FALDO ontology (36), Semanticscience Integrated Ontology (37), Sequence Ontology (38) and Relation Ontology (39) for illustrating all the information in the existing DDBJ entries and INSDC resources. The RDF version of the DDBJ annotated sequence records are available at the DDBJ FTP site (ftp://ftp.ddbj.nig.ac.jp/rdf/).

## FUTURE DIRECTION

In the present report, we introduced updates of the DDBJ datasets, data submissions, and analytical systems during the past year. We plan to develop a unified submission portal for all database systems, along with a semi-automatic annotation and curation system. The key technology is RDF, and the effort to translate DDBJ sequence records into RDF is under way.

The current focuses at DDBJ Center are as follows: (i) improved network security and data management for JGA; (ii) virtualization of computing infrastructure for better development and analysis on the HPC environment and (iii) restructuring of data processes for updating INSDC databases. In addition, to enhance research productivity on the NIG supercomputer, we are constructing an experimental system to enable not only the operation of HPC oriented software systems (MPI, grid engine) and big-data oriented systems (Spark, YARN) but also the operation of Linux containers (Docker etc.) which allow users to build, re-distribute, and run a set of analysis programs in various kinds of calculation environments.
